# The effects of acetylsalicylic acid on performance, carcass traits, breast meat quality and white striping muscle defects in broiler chickens

**DOI:** 10.1002/jsfa.14166

**Published:** 2025-02-05

**Authors:** Gülşah Güngören, Ulku Gulcihan Simsek, Alper Güngören, Mehmet Çiftçi, Hatice Eröksüz, Burak Karabulut, Yasin Baykalir, Mücahit Kahraman, İsmail Demircioğlu, Şermin Top

**Affiliations:** ^1^ Department of Animal Science, Faculty of Veterinary Medicine Kastamonu University Kastamonu Turkey; ^2^ Department of Animal Science, Faculty of Veterinary Medicine Fırat University Elazığ Turkey; ^3^ Department of Food Hygiene and Technology, Faculty of Veterinary Medicine Kastamonu University Kastamonu Turkey; ^4^ Department of Animal Nutrition and Nutritional Disease, Faculty of Veterinary Medicine Fırat University Elazığ Turkey; ^5^ Department of Pathology, Faculty of Veterinary Medicine Firat University Elazığ Turkey; ^6^ Department of Biostatistics, Faculty of Veterinary Medicine Balikesir University Balikesir Turkey; ^7^ Department of Animal Science, Faculty of Veterinary Medicine Harran University Şanlıurfa Turkey; ^8^ Department of Anatomy, Faculty of Veterinary Medicine Harran University Şanlıurfa Turkey; ^9^ Department of Animal Nutrition and Nutritional Disease, Faculty of Veterinary Medicine Harran University Şanlıurfa Turkey

**Keywords:** white striping, meat quality, irisin, aspirin, defect, VEGF

## Abstract

**BACKGROUND:**

This research aims to reduce white striping muscle defects induced by vascular inflammation and hypoxia using the anti‐inflammatory, antiplatelet and anti‐atherothrombotic properties of acetylsalicylic acid (ASA). To this end, the effects of different doses (0.3, 0.6, 1, 3 and 6 g L^−1^) of ASA added to drinking water at 24–48 days on growth performance, carcass traits, footpad dermatitis, white striping and breast meat quality parameters were investigated.

**RESULTS:**

The results indicate that 0.3, 1, 3 g L^−1^ and especially 0.6 g L^−1^ ASA treatment significantly improved growth performance and meat quality parameters. Also, doses of 0.3, 6 g L^−1^ and especially 0.6 g L^−1^ of ASA treatment reduced the incidence of white stripe muscle defects.

**CONCLUSIONS:**

Consequently, 0.6 g L^−1^ ASA treatment reduced macrophage infiltrations and myodegeneration caused by growth rate. In addition, this dose increased vascular endothelial growth factor and decreased irisin level in breast muscle. The study also shows that high doses of ASA treatment (3 and 6 g L^−1^) may make footpad dermatitis more common. This may be due to the fact that ASA can cause side effects such as gizzard ulcers and kidney damage in broiler chickens. © 2025 The Author(s). *Journal of the Science of Food and Agriculture* published by John Wiley & Sons Ltd on behalf of Society of Chemical Industry.

## INTRODUCTION

Poultry meat's health benefits, simplified preparation and economical cost have made it a popular choice among meat consumers.^1^ From the past to the present, modern production practices, such as selective breeding, feeding technologies and industrial production, have significantly reduced the time required for commercial birds to reach market‐sized weight.^1^, ^2^ In 1925, a 112‐day broiler's average market weight was 1.12 kg; yet in 2023, it reached a market weight of 2.96 kg after only 47 days.^3^ Despite the intention to increase meat yield, this fast growth unfortunately led to some unforeseen meat abnormalities.^4^ Although the mechanism by which fast growth in broilers provokes meat abnormalities is not fully understood yet, it is apparent from current literature that increased growth rates have a higher incidence of meat abnormalities such as white striping (WS).^4^, ^5^, ^6^

WS is visually characterized by the white lines of intramuscular deposits in raw meat parallel to muscle fibers, mainly in the breast. It is linked to vascular inflammation, hypoxia and the infiltration of macrophages because the pectoralis major muscle proliferates, which throws off its metabolism and homeostasis.^7^ Chicken breast products with WS do not appeal to customers because of the decreased meat quality, such as a decrease in protein content, high pH, lower drip loss, high cooking loss, negative effect on palatability and tougher taste^7^, ^8^, ^9^, ^10^ Furthermore, Kuttappan *et al*. reported that a WS phenotype was present in more than 55% of the breast meat from broilers, and that this appearance worsened with increasing slaughter age.^11^ Researchers have previously attempted to reduce the rate of WS in breast meat through feed restriction strategies, short‐term reduction of lysine, alternative methionine supplementation and dietary supplementation with antioxidants or trace elements.^6^, ^12^, ^13^, ^14^ Although dietary intervention and genetic selection strategies have tried to reduce this myopathy, only a limited number have been effective in reducing WS without impeding growth.^7^

Acetylsalicylic acid (ASA), often known as aspirin, is a kind of nonsteroidal anti‐inflammatory drug (NSAID) that reduces inflammation and has many pharmacological effects, such as antipyretic, analgesic and antiplatelet properties.^15^ ASA has been studied as an additive to feed in broilers primarily to prevent the negative impacts of heat stress and improve growth performance.^16^, ^17^, ^18^, ^19^, ^20^ Zhang *et al*. also reported that aspirin eugenol ester does not affect the growth performance and carcass yield of broilers raised under normal density stocking conditions.^21^ Similarly, Salah *et al*. did not observe a significant difference between heat‐stressed broilers fed a basal diet and heat‐stressed broilers fed an ASA‐supplemented diet in terms of carcass yield.^22^ In addition, it has been reported that ASA does not affect growth performance and carcass traits in Japanese quails under heat stress.^23^ However, some research indicated that dietary supplementation with ASA improved growth performance, increased feed intake, enhanced feed efficiency, increased the relative weight of breast and drumsticks and decreased abdominal fat and cecal *E.coli* counts in broilers under non‐stressful conditions.^17^, ^19^, ^20^, ^24^ According to researchers, ASA plays a key role in lowering cholesterol and triglycerides in blood and meat, as well as improving immune function and antioxidant enzymes. Thus, its activity results in enhanced growth performance and meat quality parameters such as post‐mortem pH, color, water‐holding capacity and cooking loss.^19^, ^20^, ^25^

Nevertheless, it should not be ignored that ASA use is associated with severe morbidity and mortality because of its adverse effects on several organs, including the liver, stomach and kidneys. The most common side effects of ASA use are the development of gastrointestinal (GI) ulcers, hepatotoxicity and nephrotoxicity.^26^, ^27^ Only a few researchers have reported that ASA may have toxic effects in broiler chickens.^28^, ^29^, ^30^

The study reported here primarily aimed to benefit from ASA's anti‐inflammatory, antiplatelet and anti‐atherothrombotic effects to help reduce the incidence of WS muscle defects resulting from vascular inflammation and hypoxia caused by fast growth in broiler chickens. Based on this hypothesis, in this research, the effects of different doses of ASA added to the drinking water of chickens during their growth phase on WS muscle defects and meat quality were investigated. In addition, the findings also revealed surprising and overlooked effects that are at odds with the findings of some current literature.

## MATERIAL AND METHODS

The research protocol was authorized by Harran University Animal Experiments Local Ethics Committee (HRÜ‐HADYEK, Şanlıurfa, Turkey) with the decision dated 05.08.2021 and numbered 01‐05.

### Housing, diets, animals and experimental design

A total of 300 one‐day‐old Ross 308 male broiler chickens (Aviagen, Newbridge, UK) were purchased from a commercial farm and enrolled into a 48‐day trial. The chicks were randomly assigned into six groups: (C) control (without treatment); (ASA1) added 0.3 g L^−1^ ASA in water; (ASA2) added 0.6 g L^−1^ ASA in water; (ASA3) added 1 g L^−1^ ASA in water; (ASA4) added 3 g L^−1^ ASA in water; (ASA5) added 6 g L^−1^ ASA in water. There were five replicates per group with each of ten birds during the experimental period. The diet was the same for all six treatment groups. The broilers were provided with unrestricted access to feed and water and used a three‐phase feeding procedure, which included a starter diet for the first 24 days of life, a grower diet from day 24 to day 35 and a finisher diet from day 36 to day 48 (supporting information, Table S1). ASA was provided in water from growth phase to end of the finisher phase (24–47 days). Diets' nutritional composition and broilers' needs were evaluated based on National Research Council (NRC) standards. The chicks were raised in cages measuring 1 m × 1 m in a mechanically ventilated room. During the first phase (days 1–5), the temperature in the feeding chamber was kept at a consistent range of 32–34 °C. Subsequently, the temperature was gradually reduced by 2 °C each week until it reached the ultimate range of 22–24 °C. The relative humidity was regularly kept within the range of 40–60%. During the first 3 days, the broilers were subjected to continuous light, which was then followed by a following program of 23 h of light and 1 h of darkness. Their growing process was closely monitored and clearly documented.

### Growth performance

Total body weight and remaining feed per pen were recorded on days 24, 30, 36, 42 and 48 in the early morning. Average body weight (ABW), average daily weight gain (ADWG), average daily feed intake (ADFI) and feed conversion ratio (FCR) were calculated.

### Footpad dermatitis

Footpad dermatitis measurements were performed as previously proposed by Rushen *et al*.^31^ The scoring of footpad dermatitis was performed by carefully examining the right and left footpads of chickens with the naked eye. Footpads were assigned a score between 0 (no lesions) and 4 (severe lesions).

### Slaughter process and carcass traits

At the end of the experiment, a total of 90 animals, 3 from each replication and 15 from each group, reflecting the replication average, were selected. The feed was withdrawn 8 h before slaughter. After the ethical slaughtering process, every carcass was scalded at 60 °C for 90 s. Following mechanical plucking (Cimuka, Turkey), the viscera, lungs, heads and feet were removed manually, and carcasses were rinsed with water to remove blood, feathers and other loose debris. The whole carcass, breasts, legs, wings, back and neck, liver, heart and spleen were weighed and calculated as a percentage of body weight.

### White striping analysis

The analysis was made according to the method specified by Russo *et al*.^32^ Briefly, the pectoralis major muscles of all broilers were exposed, and WS analysis was carried out. WS grades of 0, 1 and 2 were used, indicating normal (absent), moderate (<1 mm thick) or severe (>1 mm thick) depending on the extent of WS.

### Histopathology and immunohistochemistry

Samples were obtained from the m. pectoralis major muscle cranioventral area of 90 slaughtered chickens. About 3 cm^3^ of muscle was collected from each sample. All samples were properly labeled and stored in 10% formaldehyde. Histopathology samples were fixed, dehydrated, cleared, infiltrated, embedded in paraffin (Leica TP 1020, Wetzlar, Germany), sectioned (Leica RM2125, Wetzlar) and sliced into 5 μm thick sections then stained with hematoxylin–eosin (Leica Autostainer XL, Wetzlar, Germany). Additionally, Masson Trichrome staining was performed to determine the severity of fibrosis. The stained sections were observed under a microscope (Olympus DP72, Tokyo, Japan) to analyze semi‐qualitatively in terms of infiltration, degeneration, fibrosis, lipidosis and vascularization (0: none, 1: mild, 2: moderate, 3: severe).^33^ Also, vascular endothelial growth factor (VEGF) activity was studied using the streptavidin biotin peroxidase complex method on paraffin sections that had already been prepared. The appropriate procedure was followed using an IHC kit (Boster Biotechnology Technology, Wuhan, China) according to the manufacturer's instructions. After the sections were incubated with a suitable VEGF primary antibody, they were colored with 3‐amino‐9‐ethylcarbozole chromogen and given a counterstain of Gill's hematoxylin.^34^ Sections sealed with a water‐based adhesive were examined under a light microscope. Positive stainings were examined with a 40° objective and scored (0: none, 1: mild, 2: moderate, 3: severe).

### Determination of irisin level in pectoral muscle

Western blot techniques were utilized to measure irisin (fibronectin type III domain‐containing protein 5, FNDC5) in breast muscle tissues from 90 slaughtered chickens. For accurate value representation, three tissues from each animal in each group were homogenized and pooled. After SDS‐PAGE fractionation in a 10% running gel, the proteins were transferred to a nitrocellulose membrane under electric current in a semi‐dry environment and calorimetrically determined for antibodies and FNDC5 bands. Imaging software (NIH ImageJ, California) was used to semi‐quantitatively calculate membrane band relative density (RD). Protein samples were prepared by boiling tissues with 4× buffer, loaded into running gel at 30 μg (30 mL)^−1^ and electrophoresed at 120 V constant current for 90 min without molecular weight separation. After electrophoresis, protein samples transferred to the nitrocellulose membrane were sealed overnight in a 5% blocking solution (bovine serum albumin (BSA) + Tween20 Tris buffer (TBST)) at 4 °C. The membrane was then washed four times with TBST to remove BSA and incubated with rabbit‐produced anti‐FNDC5 antibody diluted with BSA + TBST at 1:1000 at 37 °C for 90 min. Secondary antibody anti‐rabbit IgG‐HRP diluted 1:5000 was incubated on the membrane for 60 min at 37 °C after four TBST washes. The membrane's bands formed after the final washes when HRP and 3,3‐diaminobenzidine substrate were added. To improve western blotting reliability, *β*‐actin antibodies were employed with the identical protein samples and techniques, and a control process was run. The results were normalized by comparing RD values of FNDC5 and *β*‐actin bands (FNDC5/*β*‐actin) for statistical analysis.^35^

### Meat quality parameters

#### pH analysis

Fifteen minutes after slaughter, before the plucking step, a 2.5 cm dagger electrode was placed under the surface of the breast muscle to measure the pH 15 of the carcass. Also, the pH 24 value of the breast muscle 24 h after the slaughter was measured. The probe of the standardized pH meter was stuck to three different points of the breast meat. Both pH 15 and pH 24 were recorded by taking the average of every three measurements.

#### Color analysis

After pH value measurements, color value was recorded of the skinless breast muscle using a colorimeter device according to the CIE trichromatic system, including lightness (L*), redness (a*) and yellowness (b*).^36^

#### Protein, fat, moisture and crude ash analysis

The basic nutritional composition of chicken breast meat was analyzed according to recognized Association of Analytical Chemists methods.^37^ Crude protein and intramuscular fat levels were determined by the Kjeldahl and Soxhlet extraction methods, respectively. Moisture and crude ash content were determined using a gravimetric method.

#### Cooking loss (CL) analysis

For determination of CL, chicken breast meat was weighed (initial weight, iw), put in a sealed bag and cooked in a water bath (75 °C) until the internal temperature reached 70 °C. The sample was then cooled, dried at room temperature and weighed (final weight, fw).^38^ The formula for determining cooking loss (%) is [(iw − fw)/iw] × 100.

#### Water‐holding capacity (WHC) analysis

The WHC was determined following the procedure described in Rinwi *et al*.^39^ A sample of about 10 g was wrapped in gauze and put into a 50 mL tube with absorbent cotton wool. The tube was then centrifuged at 9000 × *g* for 10 min at 4 °C. After that the sample was weighed. The WHC (%) was calculated as a percentage of weight based on measurements before and after centrifugation.

### Statistical analysis

The statistical analysis was applied after checking the homogeneity (Levene test) and normality (Shapiro–Wilk test) of the variances. An analysis of variance procedure was used to compare the groups. The Tukey HSD test was applied to compare parameters where significance was present. The Kruskal–Wallis *H* and Mann–Whitney *U* tests were used for non‐normally distributed data and discrete data where scoring was used. A Pearson correlation test was performed to determine the relationship between footpad dermatitis and WS score. The SPSS 21 computer program was used in statistical analysis. At *P* < 0.05, the difference between group averages was statistically significant.

## RESULTS AND DISCUSSION

### Growth performance, carcass traits and footpad dermatitis

The effects of ASA supplementation in water on performance parameters of growing broilers during 24–48 days of age are presented in Table 1. Adding ASA to drinking water had no effect on ABW until day 42 (*P* > 0.05). In the pre‐slaughter weighing, the ASA5 group showed similar characteristics to the control, but ABW was increased by approximately 200 g in the other groups (*P* < 0.001). Apart from this, there was a highly significant difference in FCR between the control and ASA groups (*P* < 0.001) except for ASA5 (*P* < 0.05) during treatment. The results show clear positive effects on growth performance with ASA, but no noticeable differences were observed among groups except for ASA5. These findings are consistent with literature studies demonstrating the efficacy of ASA in broiler performance.^17^, ^19^, ^20^, ^24^ The absence of the expected effect in the ASA5 group raises doubts about whether this dose could cause broilers' nephrotoxicity and digestive system issues. Nakaue *et al*. confirmed this situation long ago, reporting that 0.3–0.6% ASA added to feed may be toxic and negatively affect growth performance.^30^ Similarly, Poźniak *et al*. indicated that a dose of 400 mg kg^−1^ of ASA decreased weight gain and induced gizzard ulceration in broilers.^40^

**Table 1:** Effects of ASA added to drinking water in different doses on performance parameters in broiler chickens

	Control	ASA1	ASA2	ASA3	ASA4	ASA5	*P* value	
*Average body weight (g)*								
24	868.40 ± 13.69	869.28 ± 12.81	870.96 ± 14.05	871.84 ± 14.92	869.92 ± 14.23	871.92 ± 15.68	NS
30	1288.24 ± 20.23	1282.00 ± 21.80	1280.04 ± 22.26	1303.24 ± 21.51	1287.60 ± 18.10	1287.76 ± 16.98	NS
36	1744.56 ± 25.06	1774.52 ± 23.18	1780.20 ± 29.03	1771.62 ± 31.63	1760.56 ± 26.94	1746.28 ± 32.91	NS
42	2203.48 ± 28.21	2284.12 ± 29.61	2288.62 ± 32.59	2266.04 ± 40.00	2231.50 ± 33.09	2216.72 ± 37.77	NS
48	2547.44 ± 40.26^b^	2746.92 ± 42.46^a^	2719.66 ± 39.70^a^	2766.20 ± 50.76^a^	2762.98 ± 35.81^a^	2592.48 ± 38.48^b^	***
*Average daily weight gain (g)*							
24–30	59.98 ± 0.90	58.96 ± 1.32	58.44 ± 1.38	61.63 ± 1.44	59.67 ± 1.58	59.41 ± 1.51	NS
31–36	65.19 ± 1.05^c^	70.36 ± 1.51^ab^	71.45 ± 1.70^a^	66.91 ± 1.25^abc^	67.57 ± 1.49^abc^	65.50 ± 2.20^bc^	*
37–42	65.56 ± 1.65^b^	72.80 ± 2.15^a^	72.63 ± 1.35^a^	70.63 ± 1.75^ab^	67.28 ± 1.54^ab^	67.21 ± 2.06^ab^	*
43–48	49.14 ± 2.86^d^	66.11 ± 1.89^bc^	61.58 ± 1.90^c^	71.45 ± 2.41^ab^	75.93 ± 2.20^a^	53.68 ± 2.73^d^	***
24–48	59.97 ± 0.79^b^	67.06 ± 0.58^a^	66.02 ± 0.87^a^	67.66 ± 1.01^a^	67.61 ± 1.13^a^	61.44 ± 0.45^b^	***
*Average daily feed intake (g)*							
24–30	106.12 ± 1.13	106.77 ± 1.94	106.96 ± 1.87	105.44 ± 1.26	106.84 ± 1.69	106.56 ± 0.89	NS
31–36	124.75 ± 0.66	124.22 ± 0.23	124.28 ± 0.50	124.08 ± 0.38	123.28 ± 0.79	123.91 ± 0.54	NS
37–42	113.34 ± 1.13	114.47 ± 0.74	113.64 ± 0.85	113.35 ± 0.95	112.28 ± 0.31	113.29 ± 0.21	NS
43–48	117.21 ± 0.69	118.14 ± 0.68	117.69 ± 0.84	117.29 ± 0.89	117.41 ± 0.84	117.74 ± 0.75	NS
24–48	115.36 ± 0.42	115.90 ± 0.64	115.64 ± 0.70	115.39 ± 0.64	114.88 ± 0.58	115.36 ± 0.34	NS
*Feed conversion ratio (g feed/g gain)*							
24–30	1.77 ± 0.04	1.81 ± 0.06	1.83 ± 0.06	1.71 ± 0.05	1.80 ± 0.06	1.79 ± 0.04	NS
31–36	1.91 ± 0.04^a^	1.77 ± 0.04^bc^	1.74 ± 0.05^c^	1.85 ± 0.03^abc^	1.80 ± 0.04^abc^	1.89 ± 0.07^ab^	*
37–42	1.73 ± 0.02^a^	1.57 ± 0.04^bc^	1.56 ± 0.03^c^	1.60 ± 0.04^abc^	1.70 ± 0.04^ab^	1.69 ± 0.05^ab^	**
43–48	2.39 ± 0.08^a^	1.79 ± 0.05^c^	1.91 ± 0.06^bc^	1.64 ± 0.07^d^	1.50 ± 0.04^e^	2.19 ± 0.10^a^	***
24–48	1.92 ± 0.02^a^	1.73 ± 0.02^b^	1.75 ± 0.02^b^	1.71 ± 0.03^b^	1.70 ± 0.03^b^	1.88 ± 0.02^a^	***

Control (without treatment); (ASA1) added 0.3 g L⁻¹ ASA to water; (ASA2) added 0.6 g L⁻¹ ASA to water; (ASA3) added 1 g L⁻¹ ASA to water; (ASA4) added 3 g L⁻¹ ASA to water; and (ASA5) added 6 g L⁻¹ ASA to water. Data are shown as mean ± standard error of the mean. NS, not significant, *P* > 0.05; *P < 0.05; **P < 0.01; ***P < 0.001. Means within the same row with different letters are significantly different.

The effects of ASA supplementation in water on carcass traits and footpad dermatitis of broilers are presented in Table 2. As indicated, the effect of the treatment on live weight (*P* < 0.001) was not sufficiently reflected in carcass weight (*P* < 0.01), and carcass yield (*P* < 0.05). In addition, there was no effect on wing, leg and breast ratio (*P* > 0.05). Several previous studies agree with the present results.^21^, ^22^, ^23^, ^41^ Most researchers have reported similar effects on carcass weight and carcass yield. However, they also reported relative high weight of breasts and drumsticks.^24^, ^41^, ^42^, ^43^ Researchers attribute the weight gain to the fact that ASA increases the performance of chickens by preventing the formation of free radicals and providing protection against oxidative damage in tissue and liver cells.^40^ Also, researchers found a reduction in relative weight in the GI organs in the treated groups compared to the control group and interpreted this finding as representing a higher contribution of the breast and drumstick and a higher FCR representing better health status.^20^, ^24^ Contrary to the interpretations above, in cases of aspirin‐induced GI ulcers, intestinal emptying will be rapid, feed intake will decrease and weight gain will be reduced.^44^ The fact that the control and ASA5 groups showed similar FCR values in this study supports the hypothesis that dose‐dependent aspirin‐induced GI ulcers may occur. These controversial and unexpected results may be related to the dose and method of administration of ASA.

**Table 2:** Effects of ASA added to drinking water in different doses on carcass traits and footpad dermatitis in broiler chickens

Carcass trait	Control	ASA1	ASA2	ASA3	ASA4	ASA5	*P* value
Live weight (g).	2522.20 ± 53.38^b^	2722.73 ± 96.72^a^	2684.43 ± 81.23^a^	2738.20 ± 90.13^a^	2726.07 ± 82.34^a^	2538.33 ± 80.81^b^	***
Carcass weight (g)	1934.00 ± 41.43^b^	2048.00 ± 79.29^ab^	2047.33 ± 64.95^ab^	2132.67 ± 75.25^a^	2110.00 ± 64.42^a^	1949.33 ± 64.03^b^	**
Carcass yield (%)	76.39 ± 0.48^b^	78.06 ± 1.09^ab^	77.89 ± 0.47^ab^	79.50 ± 0.57^a^	78.13 ± 1.12^ab^	77.31 ± 0.41^ab^	*
Breast (% of carcass weight)	35.24 ± 0.46	35.80 ± 0.64	35.75 ± 0.51	36.01 ± 0.65	36.02 ± 0.79	35.32 ± 0.62	NS
Whole leg (% of carcass weight)	41.71 ± 0.27	42.40 ± 0.64	42.16 ± 0.39	42.86 ± 0.41	42.27 ± 0.75	41.82 ± 0.47	NS
Wings (% of carcass weight)	10.00 ± 0.22	10.12 ± 0.21	10.15 ± 0.18	10.42 ± 0.19	10.33 ± 0.23	10.02 ± 0.13	NS
Back‐neck (% of carcass weight)	11.76 ± 0.26	11.96 ± 0.25	11.82 ± 0.27	12.15 ± 0.28	12.17 ± 0.21	11.75 ± 0.27	NS
Liver (%)	2.07 ± 0.09^ab^	2.22 ± 0.14^ab^	2.26 ± 0.05^ab^	2.38 ± 0.08^a^	2.28 ± 0.15^ab^	2.01 ± 0.11^b^	*
Heart (%)	0.70 ± 0.04^b^	0.74 ± 0.06^ab^	0.74 ± 0.04^ab^	0.85 ± 0.04^a^	0.80 ± 0.05^ab^	0.68 ± 0.03^b^	*
Spleen (%)	0.10 ± 0.01	0.10 ± 0.01	0.10 ± 0.01	0.10 ± 0.01	0.10 ± 0.01	0.10 ± 0.01	NS
Footpad dermatitis	1.89 ± 0.22^c^	3.06 ± 0.16^ab^	2.94 ± 0.14^ab^	2.48 ± 0.15^bc^	2.51 ± 0.19^bc^	3.35 ± 0.15^a^	***

Control (without treatment); (ASA1) added 0.3 g L⁻¹ ASA to water; (ASA2) added 0.6 g L⁻¹ ASA to water; (ASA3) added 1 g L⁻¹ ASA to water; (ASA4) added 3 g L⁻¹ ASA to water; and (ASA5) added 6 g L⁻¹ ASA to water. Data are shown as mean ± standard error of the mean. NS, not significant, *P* > 0.05; *P < 0.05; **P < 0.01; ***P < 0.001. Means within the same row with different letters are significantly different.

Furthermore, this research revealed that the ASA5 group had the greatest level of footpad dermatitis, whereas the control group had the lowest level (*P* < 0.001). The incidence of footpad dermatitis was relatively high in the ASA3 and ASA4 groups, similar to the control group (*P* > 0.05). ASA is known to change kidney functions, increase urination–defecation and acidify urine.^26^, ^45^, ^46^, ^47^ Therefore, according to the results of this study, ASA treatment with water may increase the incidence of footpad dermatitis in broiler chickens.

This study found no significant relationship between footpad dermatitis and the ratio of WS myopathies (*P* > 0.05). There is a study in the literature reporting that a poor gait score may be associated with another type of myopathy, namely wooden breast myopathy.^48^ The relationship between broiler breast myopathies and gait score and footpad dermatitis requires more detailed research.

### White striping, histopathology, immunohistochemistry and irisin level in pectoral muscle

**Figure 1:**
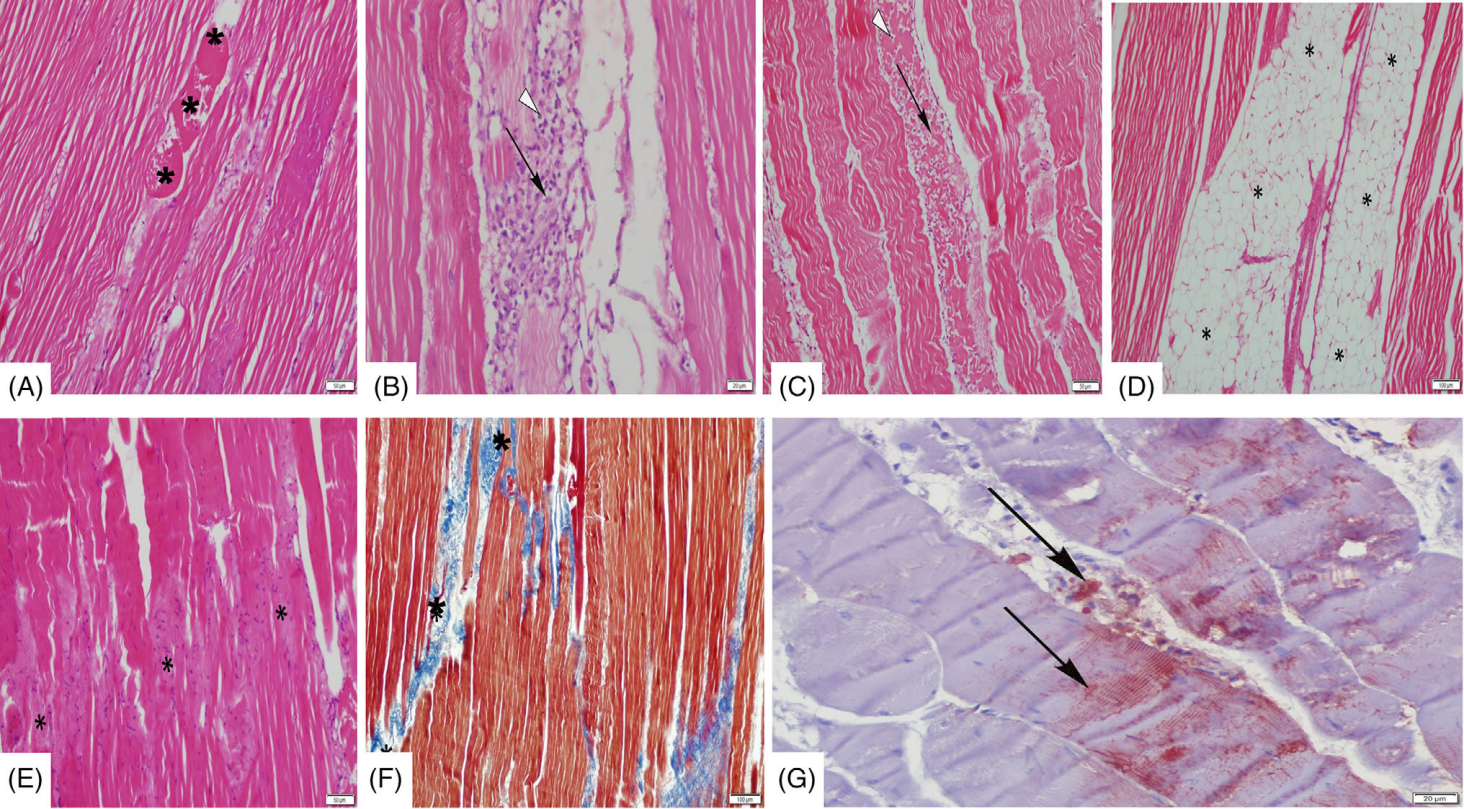
Photomicrographs of hematoxylin–eosin‐stained pectoral muscle tissue from control and treatment groups showed that the muscle tissue had white stripes and was reactive with VEGF. (A) Necrotic muscle fibers (*). (B, C) Interstitial mononuclear cell infiltrates. Macrophages (arrow) and lymphocytes (arrowhead) are present in the necrotic area, along with accompanying cellular infiltration. (D) Area with a lot of fat infiltration (*). (E, F) Fibrosis in the muscle fibers and the blue‐stained areas corresponding to Masson Trichrome staining. (G) VEGF immunoreactivity in muscle fibers and interstitial vascular area.

**Figure 2:**
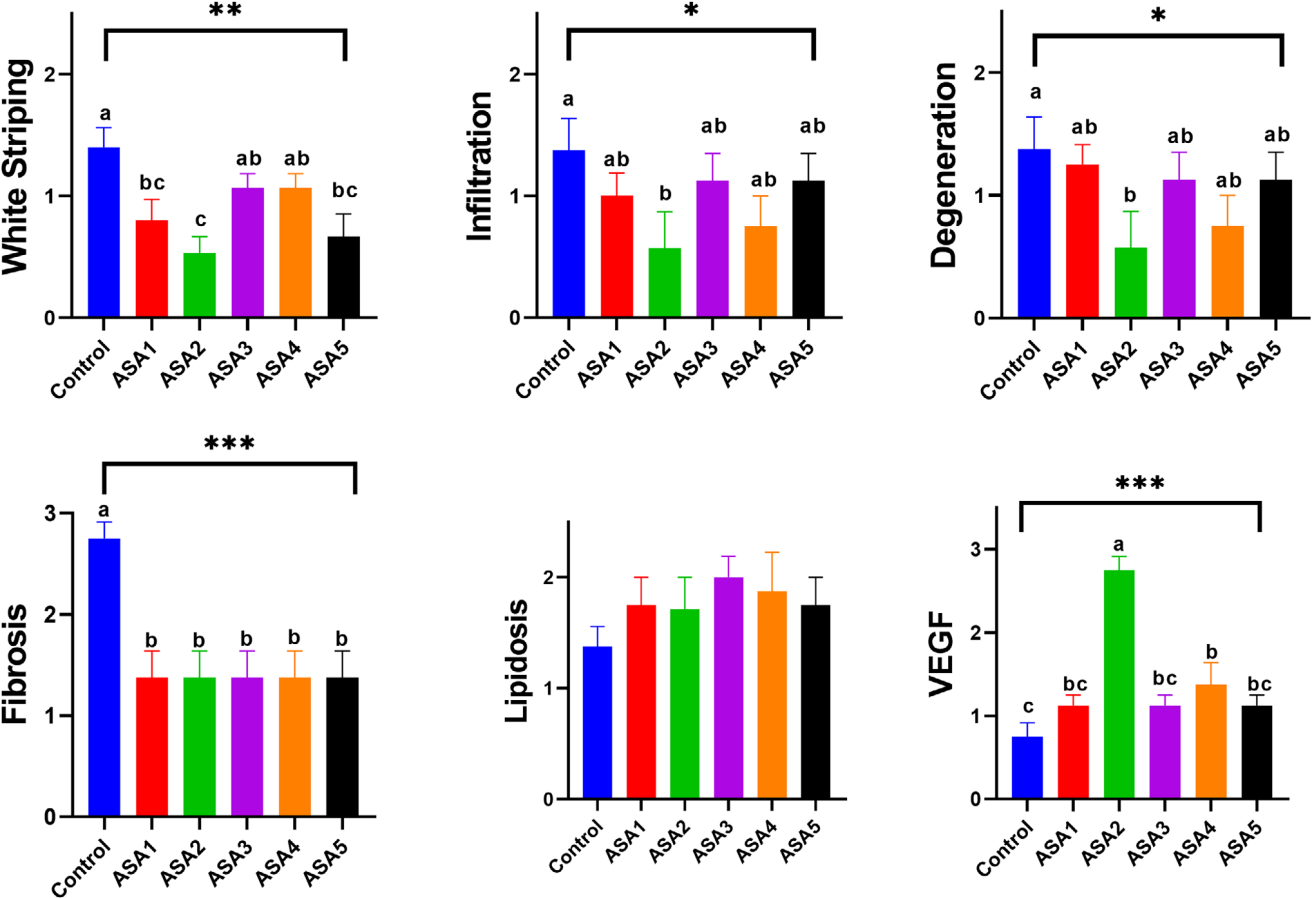
Effect of ASA treatment in drinking water on broiler breast meat WS, infiltration, degeneration, fibrosis, lipidosis and VEGF score. Control (without treatment); (ASA1) added 0.3 g L^−1^ ASA to water; (ASA2) added 0.6 g L^−1^ ASA to water; (ASA3) added 1 g L^−1^ ASA to water; (ASA4) added 3 g L^−1^ ASA to water; and (ASA5) added 6 g L^−1^ ASA to water. The values represent mean and standard error. Different letters (a, b, c) on the bars indicate statistically significant differences. *P < 0.05; **P < 0.01; ***P < 0.001.

Several researchers have linked WS myopathies to rapid muscle growth, insufficient vascularization, hypoxia, lipidosis, dysregulation of calcium and oxidative stress that may lead to tissue degeneration.^9^, ^49^, ^50^, ^51^, ^52^, ^53^ Also, Kuttappan *et al*. had previously described histopathological changes in broilers' white striped pectoral muscles.^54^ In this study, necrosis, mononuclear cell infiltration, lipidosis, interstitial inflammation and fibrosis were histopathologically confirmed in the white striped pectoral muscle tissue (Fig. 1). The effects of ASA supplementation in water on WS and histopathology scores are shown in Fig. 2. The control group had the greatest incidence of WS myopathy (*P* < 0.01), infiltration (*P* < 0.05) and degeneration (*P* < 0.05) in the breast muscles, whereas the ASA2 group had the lowest incidence. ASA has a wide spectrum of pharmacological effects, which include prostaglandin‐dependent and ‐independent actions. It is known that low‐dose ASA can significantly inhibit cyclooxygenase (COX) in skeletal muscle and reduce the inflammatory, skeletal muscle health regulator PGE_2_.^55^, ^56^, ^57^ Also, ASA has been found to increase calcium influx at low concentrations, while suppressing influx at high concentrations in cells. Briefly, low dose of ASA rectifies calcium homeostasis, decreases reactive oxygen species and increases nitric oxide production.^58^, ^59^ Nitric oxide plays important roles in regulation of mitochondrial function and improves mitochondrial biogenesis of skeletal muscle.^60^ Aspirin also acetylates lysine residues in fibrinogen, which directly promotes fibrinolysis.^61^ This explains the low incidence of WS, infiltration and degeneration in ASA2 treatment. According to the results of the current research, the control group had the lowest levels of VEGF, as a pro‐angiogenic factor, while ASA2 demonstrated the highest levels (*P* < 0.001). Angiogenesis is the process through which new capillaries sprout from pre‐existing ones. Many studies indicate that ASA has anti‐angiogenesis effects, including inhibition of hypoxia‐reoxygenation signaling pathways and VEGF.^62^, ^63^ Unlike NSAIDs like ibuprofen, ASA inhibits platelet aggregation through irreversible COX acetylation, resulting in nearly complete suppression of thromboxane A2 production by platelets. This pharmacodynamic effect continues throughout a platelet's lifespan.^63^, ^64^ Contrary to the literature, the findings of this study support ASA's VEGF‐mediated angiogenic effect in broilers. In fact, poor antiplatelet effects were reported in human patients after ASA treatment, a phenomenon reported as aspirin resistance.^65^ Another possible explanation of this situation is the possibility of the production of reactivation proteins in avian platelets compared to mammalian platelets. Avian platelets, which have a nucleus, can produce proteins required for COX reactivity after exposure to aspirin, just like vascular endothelial cells.^61^, ^66^, ^67^

**Figure 3:**
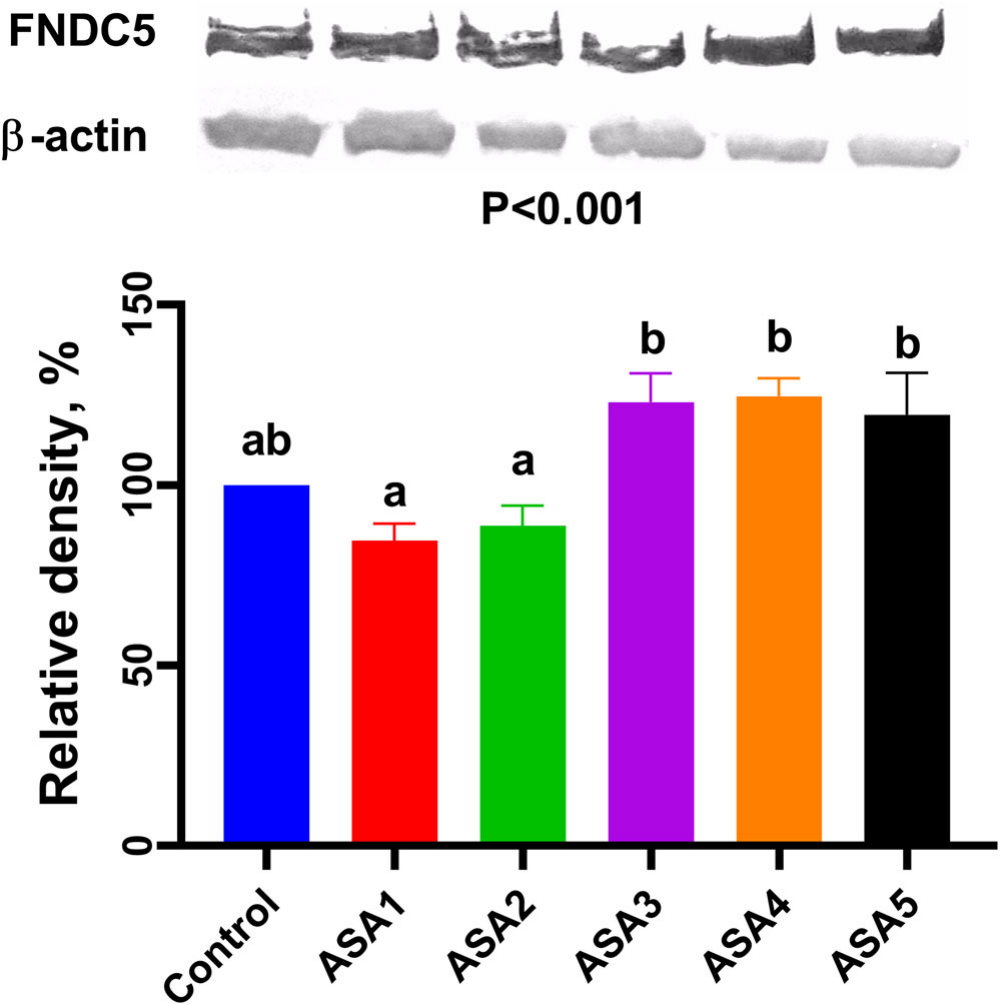
Effect of ASA treatment in drinking water on broiler breast meat irisin (FNDC5) density. Control (without treatment); (ASA1) added 0.3 g L^−1^ ASA to water; (ASA2) added 0.6 g L^−1^ ASA to water; (ASA3) added 1 g L^−1^ ASA to water; (ASA4) added 3 g L^−1^ ASA to water; and (ASA5) added 6 g L^−1^ ASA to water. The values represent mean and standard error. Different letters (a, b) on the bars indicate statistically significant differences.

Irisin is a myokine that is associated with increased energy expenditure. Irisin is mainly formed by muscle tissue through the Ca^2+^–adenosine 5′‐monophosphate‐activated protein kinase (AMPK)–peroxisome proliferator‐activated receptor *γ* coactivator‐1*α* (PGC‐1*α*)–FNDC5 pathway. It stimulates the production of myoblasts by activating the extracellular signal‐related kinase 1/2 (ERK1/2) and IL‐6 pathways in a way that affects the same cells, which is important for regulating muscle growth and proliferation.^68^, ^69^ The effects of ASA supplementation in water on irisin content in broiler pectoral muscle tissue are demonstrated in Fig. 3. When the relative density of irisin in breast muscle samples was at 100% in the control group, the ASA1 group showed a 16% decrease in FNDC5 level compared to the control, and the ASA2 group showed a 14% decrease. On the contrary, the ASA3, ASA4 and ASA5 groups showed an increase of 21%, 23% and 16%, respectively (*P* < 0.001). Irisin has been shown to regulate glucose and lipid homeostasis. In general, FNDC5 is highly expressed in skeletal muscle and adipose tissue.^70^ Moreover, in low‐dose ASA groups, the decrease in irisin density is an indication that intramuscular adipose tissue is also decreased in pectoral muscle. As mentioned, WS is linked to accumulation of lipids, increased collagen content and crude fat content in breast muscle.^7^ There is evidence that high levels of irisin in muscle increase collagen gene expression and collagen amount.^71^, ^72^ This is confirmed by WS data in the current research. Nevertheless, the incidence of WS was decreased, whereas the concentration of irisin increased in the ASA5 group. This was an inconsistency that could link to ASA‐induced GI problems. Some experimental findings indicate that irisin has a potent therapeutic potential for intestinal ischemia/reperfusion.^73^ It may be theorized that irisin enters into biochemical reactions to eliminate GI damage, which is the primary concern, rather than muscle tissue recovery.

### Breast meat quality

**Figure 4:**
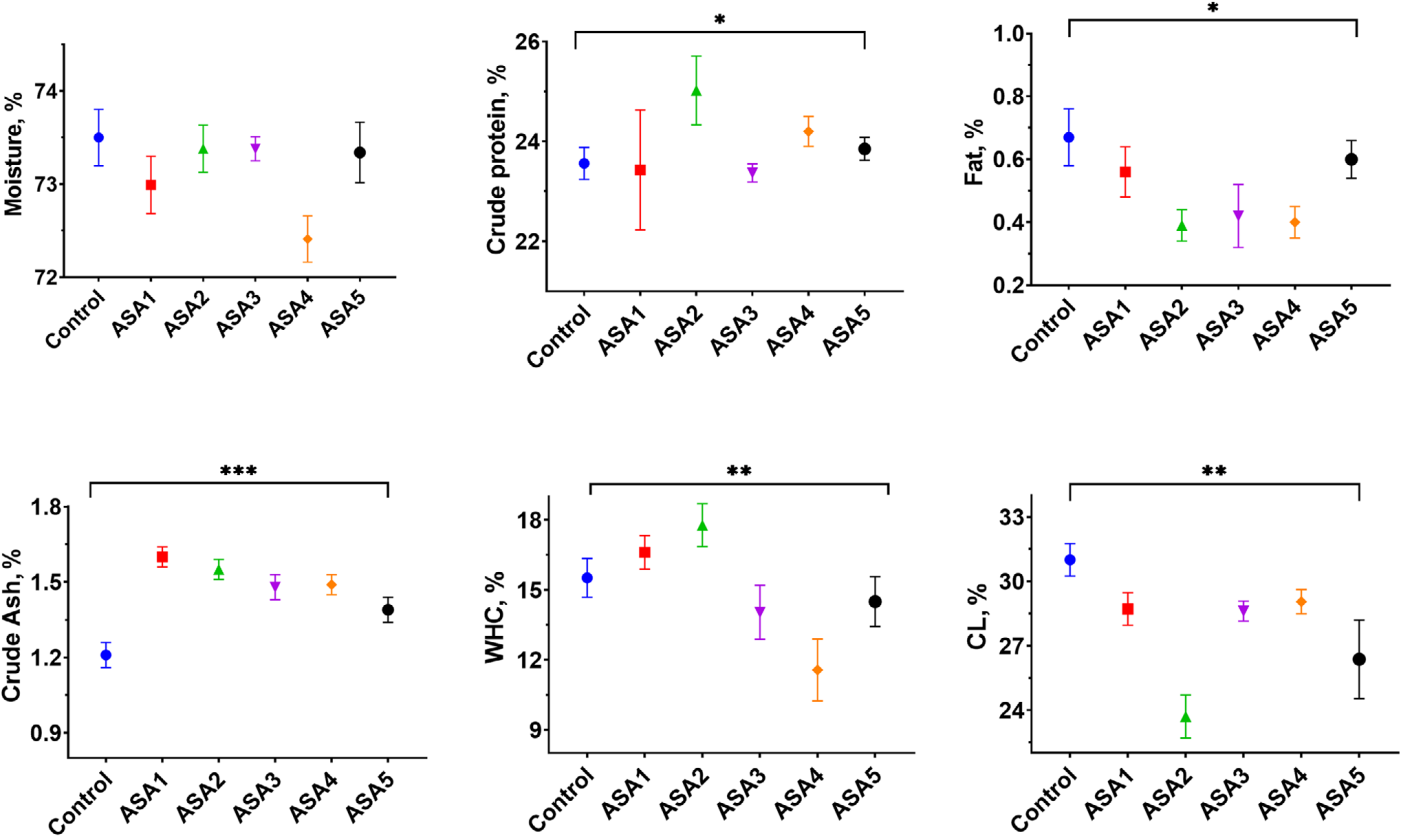
Effect of ASA treatment in drinking water on broiler breast meat quality. Control (without treatment); (ASA1) added 0.3 g L^−1^ ASA to water; (ASA2) added 0.6 g L^−1^ ASA to water; (ASA3) added 1 g L^−1^ ASA to water; (ASA4) added 3 g L^−1^ ASA to water; and (ASA5) added 6 g L^−1^ ASA to water. The values represent mean and standard error. *P < 0.05; **P < 0.01; ***P < 0.001.

WS can change the proximate composition of breast meat, affecting the nutritional value of breast meat products, which could trigger consumer concerns.^4^, ^6^, ^7^ This study examined the effect of ASA on WS appearance, especially in breast meat. Dry matter, crude protein, intramuscular fat, crude ash, WHC and CL data for the breast muscle of the groups are shown in Fig. 4. The moisture level in the breast fillets of the control group and ASA groups ranges from 72.4 to 73.5 (*P* > 0.05). Several studies, including the present one, indicate that there is no difference in the amount of moisture, even in samples with severe WS.^74^, ^75^, ^76^ WS breast meat indicates higher fat content and lower protein content in comparison to normal meat.^5^, ^6^, ^74^ In this study, the ASA2 group with the lowest WS score had higher protein and lower fat contents than the control group with the highest WS score (*P* < 0.05). The breast muscles affected by moderate and severe WS had a decreased ash content.^75^, ^77^, ^78^ Similarly, in this study, the lowest ash content was found in the control group with the highest WS score. Burning organic materials such as fat, which contains only carbon, oxygen and hydrogen, releases carbon dioxide and water vapor. These substances have no effect on crude ash. It is possible that the amount of ash decreases as the fat content increases in breast meat with WS.

The WHC has a direct impact on meat quality. An increase in muscle water content improves tenderness, juiciness, firmness and appearance, resulting in improved meat quality and economic value.^1^, ^79^ It has previously been reported that WS reduces the WHC and protein functionality of chicken breast meat, but the effects of WS on these properties are not uniform throughout breast muscle.^80^ In the current research, ASA2 had greatest WHC value, while increases in ASA levels reduced WHC in breast muscles. The severe degeneration of the muscle tissue in WS would explain the poor WHC. Furthermore, this reduced WHC may result from compromised renal or hepatic function due to ASA's adverse effects. The denaturation of proteins and the breakdown of cell membranes during the cooking process result in a reduction of nourishing fluids, a phenomenon known as CL. In this study, the ASA2 group had a lower CL value than the control group (*P* < 0.01). As previously stated, WS breast meat has a higher CL value than normal meat.^77^, ^79^, ^80^

**Figure 5:**
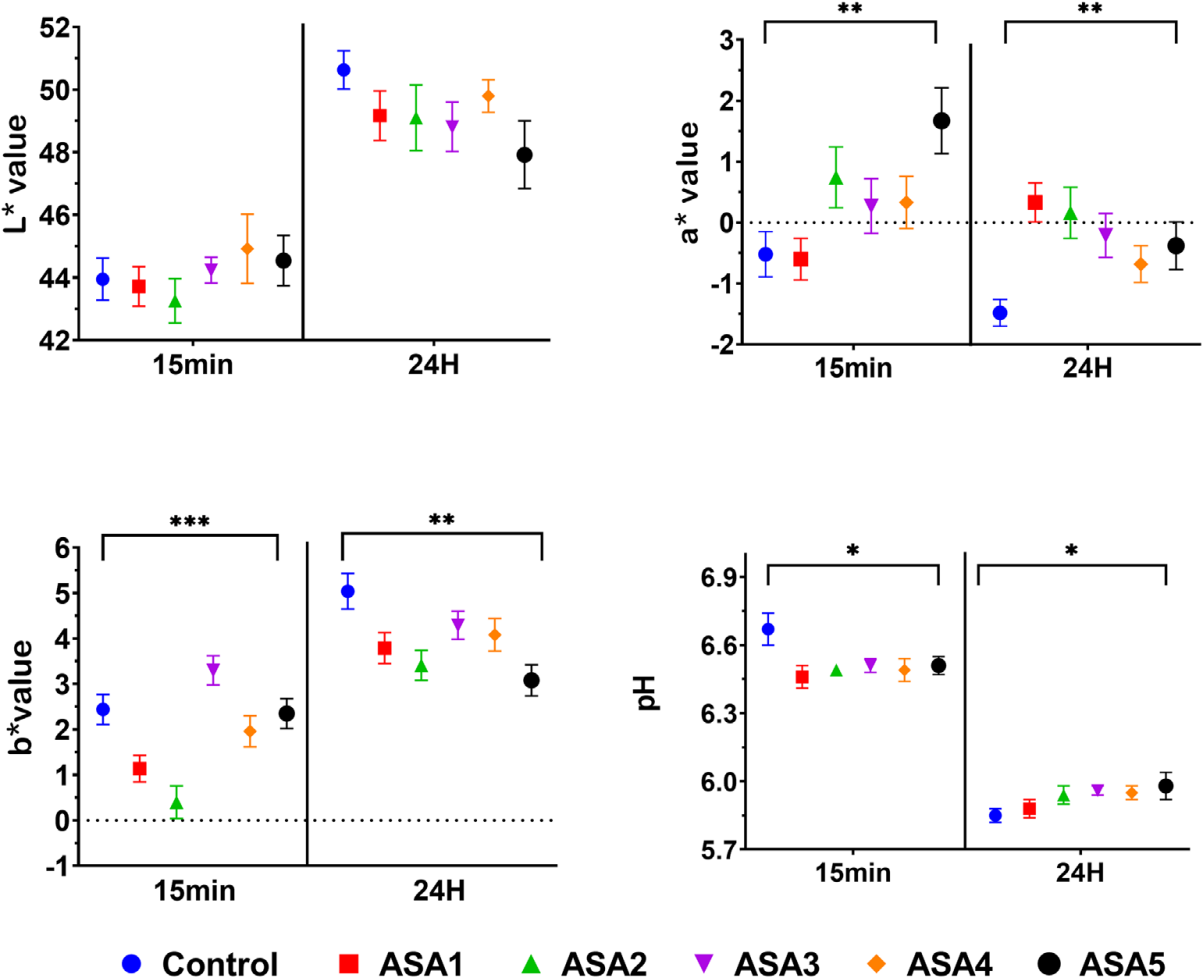
Effect of ASA treatment in drinking water on broiler breast meat L* (lightness), a* (redness/greenness) and b* (yellowness/blueness) color parameters and pH values of samples before (15 min after slaughter) and after (24 h after slaughter) rigor. Control (without treatment); (ASA1) added 0.3 g L^−1^ ASA to water; (ASA2) added 0.6 g L^−1^ ASA to water; (ASA3) added 1 g L^−1^ ASA to water; (ASA4) added 3 g L^−1^ ASA to water; and (ASA5) added 6 g L^−1^ ASA to water. The values represent mean and standard error. *P < 0.05; **P < 0.01; ***P < 0.001.

Figure 5 shows the L* (lightness), a* (redness/greenness) and b* (yellowness/blueness) color parameters and pH value of the samples before (15 min after slaughter) and after (24 h after slaughter) rigor. The pH, which was around 6.5 for all groups after slaughter, dropped below 6.0 in the post‐rigor (pH 24) phase, as expected. However, pH 24 values were higher in the ASA treatment groups than in the control group. Petracci *et al*. noted that WS breast meat has a slightly higher pH 24 value compared to normal meat, which is inconsistent with our results.^81^ This may be related to differences in the slaughter method, slaughter conditions and post‐slaughter storage conditions. The samples showed no change in the lightness (L*) value (*P* > 0.05), but ASA groups in the post‐rigor phase showed an increase in redness (a*) and a decrease in yellowness (b*) in breast meat compared to the control group (*P* < 0.01). Several researches have shown that increased WS score leads to an increase in the b* value and a reduction in the a* value of the breast meat.^5^, ^81^, ^82^ Here, it is clear that increasing the amount of adipose tissue and collagen increases the yellowness (b*) index but does not change or decrease the redness (a*) in breast meat.^5^ ASA application reduced the incidence of WS, the amount of intramuscular fat and the yellowness index. However, we have observed that applying ASA significantly increases the redness index (*P* < 0.01). Increased blood flow to tissues could raise the redness index. This rise could be due to ASA's antiplatelet effect.^66^

## CONCLUSIONS

On a daily basis, the use of low‐dose (0.6 g L^−1^) ASA or aspirin in drinking water significantly improved breast meat quality parameters, as well as reducing macrophage infiltrations and myodegenerations caused by growth rate. In addition, low‐dose (0.6 g L^−1^) ASA can increase VEGF and decrease irisin levels in breast muscle. Moreover, it can reduce the incidence of WS and promote growth performance. Nevertheless, the prolonged and increased doses of ASA (3 and 6 g L^−1^) supplementation in water did not produce the desired effects in broilers. These doses may impair the quality of the meat in fast‐growing broiler chickens' carcass quality, and make footpad dermatitis more common.

## CONFLICT OF INTEREST

The authors declare that they have no known competing financial interests or personal relationships that could have appeared to influence the work reported in this paper.

## Supporting information


**Table S1.** Ingredients, chemical composition, and energy of the diets used during the starter period (1 to 23 days of age), grower period (24 to 35 days of age) and finisher period (36 to 48d of age).

## Data Availability

The data that support the findings of this study are available from the corresponding author upon reasonable request.
