# Supplementary material for: The effects of acetylsalicylic acid on performance, carcass traits, breast meat quality and white striping muscle defects in broiler chickens
Source: J Sci Food Agric. 2025 Feb 5;105(8):4338–48. doi: 10.1002/jsfa.14166 (PMC12082017; doi:10.1002/jsfa.14166)
Supplement: Supplementary file 1 — Table S1. Ingredients, chemical composition, and energy of the diets used during the starter period (1 to 23 days of age), grower period (24 to 35 days of age) and finisher period (36 to 48d of age). [file JSFA-105-4338-s001.docx]

Table S1. Ingredients, chemical composition, and energy of the diets used during the starter period (1 to 23 d of age), grower period (24 to 35 d of age) and finisher period (36 to 48d of age).

| **Basal diet ingredients** | **Starter period**  **(1-23)** | **Grower period**  **(24-35)** | **Finisher period (36-48)** |
| --- | --- | --- | --- |
| Corn | 44.85 | 60.29 | 63.53 |
| Soybean meal (44% crude protein) | 38.00 | 28.34 | 24.20 |
| Wheat bran (Razmol) | 6.00 | 4.00 | 5.00 |
| Vegetable oil | 6.90 | 4.21 | 4.15 |
| Dicalcium Phosphate | 1.70 | 1.33 | 1.33 |
| Ground Limestone | 1.25 | 0.97 | 0.89 |
| DL- Methionine | 0.25 | 0.25 | 0.25 |
| L- Lysine hydrochloride | 0.10 | 0.16 | 0.20 |
| L- Threonine | 0.08 | 0.08 | 0.08 |
| Salt | 0.45 | 0.35 | 0.35 |
| Vitamin and mineral supplements***** | 0.50 | 0.20 | 0.20 |
| **Nutritional Composition, (%)**** |  |  |  |
| Dry matter | 90.50 | 90.00 | 89.90 |
| Cude protein | 23.00 | 19.50 | 18.00 |
| Ether Extract | 2.86 | 6.07 | 6.08 |
| Ash | 5.83 | 5.51 | 5.25 |
| Crude fiber | 2.88 | 2.73 | 2.72 |
| Calcium | 0.98 | 0.78 | 0.74 |
| Available phosphorous | 0.48 | 0.39 | 0.38 |
| Sodium | 0.21 | 0.17 | 0.17 |
| chlorine | 0.31 | 0.25 | 0.25 |
| Methionine+Cystine | 0.75 | 0.90 | 0.85 |
| Lysine | 1.36 | 1.15 | 1.07 |
| Threonine | 0.80 | 0.80 | 0.73 |
| tryptophan | 0.33 | 0.26 | 0.23 |
| ME, kcal/kg**^**^** | 3202 | 3200 | 3225 |

NA: Not available. *: Vitamin-mineral premix supplied per kg: Vitamin A, 12000 IU; Vitamin D3, 3000 IU; Vitamin E, 30 mg; Manganese, 80 mg; Iron, 60 mg; Zinc, 60 mg; Copper, 5 mg; Iodine, 1.5 mg; Cobalt, 0.3 mg; Selenium 0.15 mg

**: Calculated
